# Rhodium(i)-catalyzed asymmetric [4 + 2] cycloaddition reactions of 2-alkylenecyclobutanols with cyclic enones through C–C bond cleavage: efficient access to *trans*-bicyclic compounds†
†Electronic supplementary information (ESI) available. CCDC 1575240. For ESI and crystallographic data in CIF or other electronic format see DOI: 10.1039/c7sc04784c


**DOI:** 10.1039/c7sc04784c

**Published:** 2018-01-08

**Authors:** Xinxin Zheng, Rui Guo, Guozhu Zhang, Dayong Zhang

**Affiliations:** a Institute of Pharmaceutical Science , China Pharmaceutical University , Nanjing , P. R. China . Email: cpuzdy@163.com; b State Key Laboratory of Organometallic Chemistry , Center for Excellence in Molecular Synthesis , Shanghai Institute of Organic Chemistry , Chinese Academy of Sciences , 345 Lingling Road , Shanghai 200032 , P. R. China . Email: guozhuzhang@sioc.ac.cn

## Abstract

We report a rhodium-catalyzed asymmetric formal intermolecular [4 + 2] cycloaddition reaction of 2-alkylenecyclobutanols with α,β-unsaturated cyclic ketones leading to synthetically useful *trans*-bicyclic molecules. Three consecutive stereogenic centers are formed in a highly enantio- and diastereoselective manner. Stepwise C–C bond cleavage and annulation are likely involved in the reaction pathway. Here, iPr-Duphos is the viable chiral ligand that promotes excellent enantio-control.

## Abstract 2

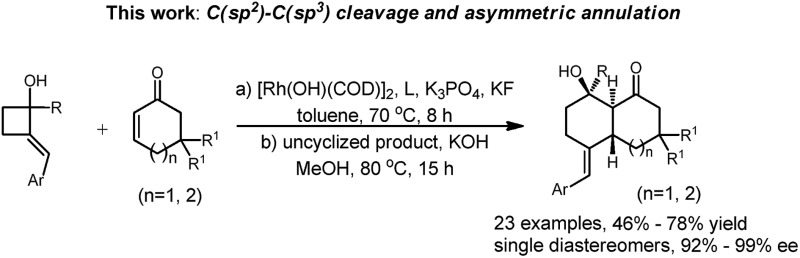
We report a rhodium-catalyzed asymmetric formal intermolecular [4 + 2] cycloaddition reaction of 2-alkylenecyclobutanols with α,β-unsaturated cyclic ketones leading to synthetically useful *trans*-bicyclic molecules.

## Introduction

**Fig. 1:**
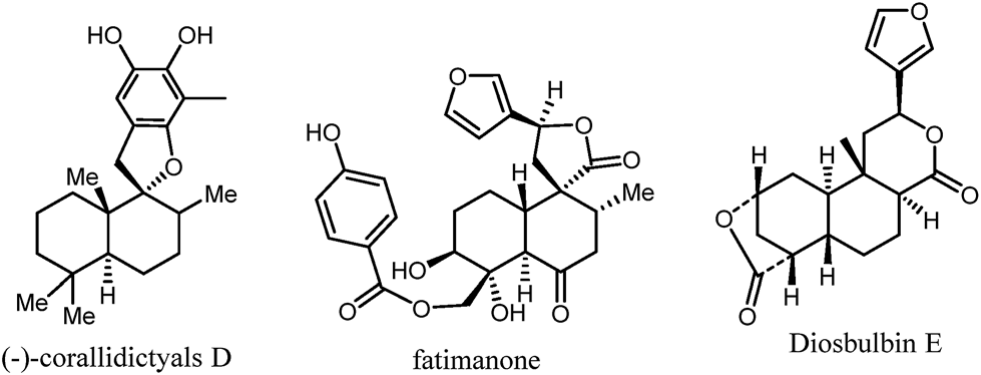
Representative of the natural products.

Bicyclic rings are found in the skeletons of many terpenoid natural products such as (–)-corallidictyals, fatimanone, and diosbulbin E (Fig. 1).^1^ Terpenoids and synthetic small molecules containing bicyclic ring structures exhibit a wide range of important bioactivities.^2^ Intermolecular [4 + 2] cycloaddition to the C2–C3 positions of α,β-unsaturated cyclic ketones has high synthetic potential for the synthesis of structurally diverse and complex bicyclic systems.^3^ Among these, the Diels–Alder (DA) reaction constitutes one of the most widely used and efficient approaches.^4^ However, DA adducts generally possess *cis* configurations that are less common in natural products; meanwhile, asymmetric catalysis has had only limited success.^5^ Therefore, new catalytic methods for the expedient synthesis of bicyclic motifs in a *trans*- and enantioselective fashion are highly desirable.

Studies of transition-metal-catalyzed selective cleavage of carbon–carbon single bonds as the initiation for further functionalizations have grown considerably in recent years due to the high potential of this strategy in synthesis.^6^ Cyclobutenols and cyclobutanols are privileged building blocks in this field.^7^ Murakami pioneered a series of studies on the rhodium-catalyzed tandem C–C single bond cleavage/formal cycloaddition of benzocyclobutenols with various functionalities including alkynes,7e,q vinyl ketones,7*j* carbene precursors,7*l* and allenes.7*f* As a special surrogate for benzocylobutenols, 2-alkylenecyclobutanols have attracted much less attention in the C–C bond cleavage research field.^8^ Therefore, the means to obtain 2-alkylidene cyclobutanols with similar reactivities of selective C–C bond cleavage and annulation would offer a new avenue to this rapidly expanding synthetic tool box eqn (1).
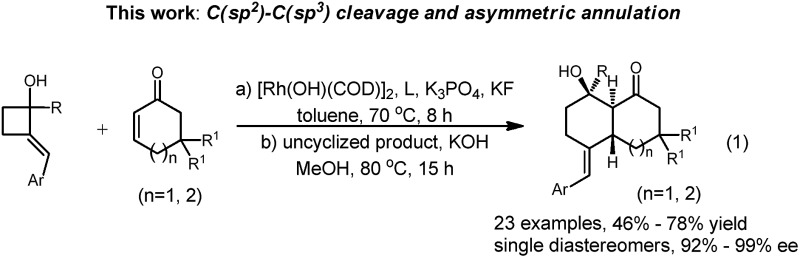


Inspired by Murakami’s work on the successful cycloaddition of benzocyclobutenol with acyclic alkyl vinyl ketones leading to tetralin skeletons,7*j* here we explore the feasibility of combining cyclic enones with 2-alkylenecyclobutanols and possible enantioinductions enabled by a proper chiral ligand. Thus, we report the highly efficient rhodium(i)-catalyzed formal [4 + 2] cycloaddition of 2-alkylene cyclobutanols with α,β-unsaturated cyclic ketones *via* a tandem C(sp^2^)–C(sp^3^) bond cleavage and cycloaddition leading to complex *trans*-bicyclic ring systems. Here, iPr-Duphos is the most effective chiral ligand to enable enantioselective transformation.

We began our studies by exploring the reaction of cyclohex-2-enone with (*E*)-2-benzylidene-1-phenylcyclobutanol. After numerous trials, the use of [Rh(COD)OH]_2_ catalyst and K_3_PO_4_ as the base produced the desired product **2a** as a single diastereomer plus uncyclized **3a** in a ratio of 1/1. Interestingly, **3a** could be separated and converted to **2a** by treatment with KOH in MeOH at 80 °C in 70% isolated yield as a single diastereomer, indicating that cyclization is highly stereospecific (Table 1, entry 1).^9^

**Table 1:** Optimization of the reaction conditions for rhodium(i)-catalyzed tandem ring opening and cyclization

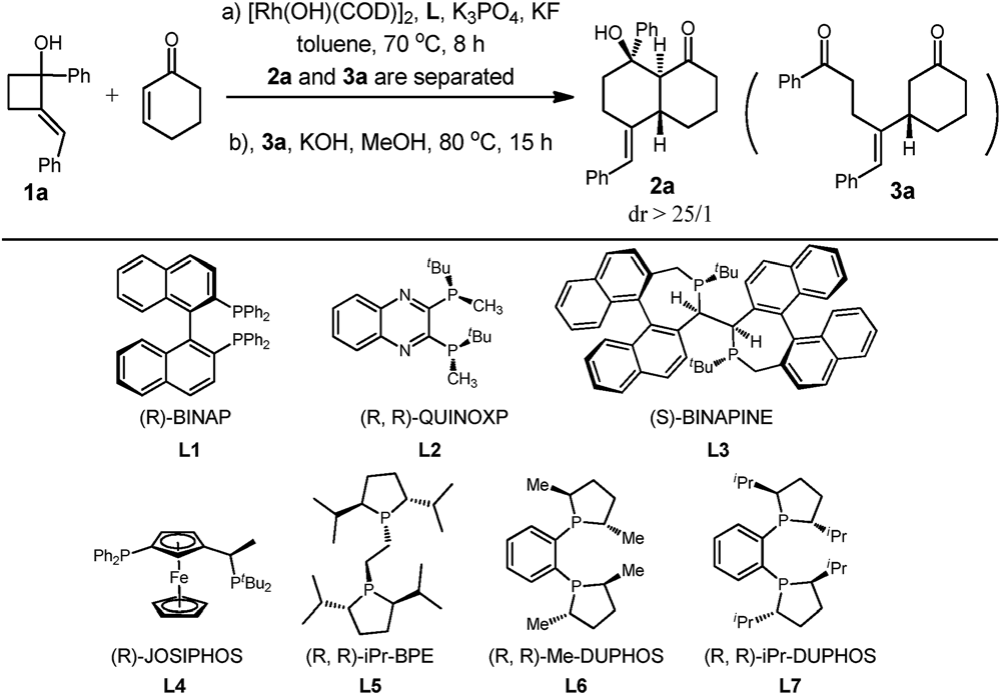				
Entry^*a*^	**L**	**2a**/**3a**	Yield (**2a**)^*b*^ [%]	ee^*c*^ [%]
1^*d*^	—	1.0/1.0	30	—
2	—	2.1/1.0	60	—
3^*e*^	—	0/1.0	49	—
4	**L1**	—	—	—
5	**L2**	1.0/1.0	30	0
6	**L3**	1.0/2.0	43	27
7	**L4**	—	—	—
8	**L5**	1.2/1.0	28	87
9	**L6**	1.2/1.0	27	96
10	**L7**	1.9/1.0	60	96

^*a*^Unless otherwise noted, two-step reactions were carried out: step a, **1a** (0.2 mmol), cyclohexenone (2 equiv.), [Rh(COD)OH]_2_ (2.5 mol%), **L** (10 mol%), K_3_PO_4_ (2 equiv.), and KF (2 equiv.) were heated in toluene (0.2 M) at 70 °C for 8 h; step b, **3a** (isolated from step a) and KOH (1.1 equiv.) were heated in MeOH (0.1 M) at 80 °C for 15 h.

^*b*^The combined yield of the two steps.

^*c*^The absolute configuration of the product was assigned by single crystal X-ray analysis of **2a**.

^*d*^Without KF.

^*e*^The reaction conditions for step a were: **1a** (0.2 mmol), cyclohexenone (2 equiv.), [Rh(COD)OH]_2_ (2.5 mol%), K_2_CO_3_ (1.1 equiv.), and 10% H_2_O in toluene (0.2 M) heated at 70 °C for 8 h.

**Fig. 2:**
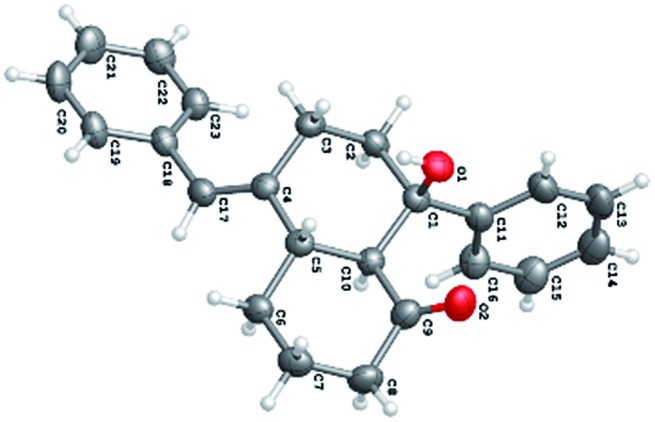
X-ray crystal structure of ***ent*-2a**.

To improve the yield of **2a**, a range of additives was tested, and to our delight 60% yield of **2a** was attained in the presence of KF, which likely facilitates the formation of enolate and the next aldol cyclization reaction (Table 1, entry 2).^10^ However, no further improvement in the yield of **2a** or in the ratio of **2a** over **3a** was obtained after many experiments. We have emphasized the use of commercial chiral ligands for asymmetric carbon–carbon bond formation. Several representative phosphine ligands such as BINAP, QuinoxP*, Binapine, and Josiphos were ineffective at catalyzing the reaction. They resulted in either low yields or negligible enantiomer ratios of **2a** (Table 1, entries 4 to 7). To our delight, a much improved enantioselectivity of 87% ee was obtained for **2a** with **L5** as the ligand (Table 1, entry 8). Further studies identified **L7** as the most effective ligand of those tested—it resulted in **2a** being obtained in 96% ee (Table 1, entry 10). Notably, the ee values of **3a** and **2a** are almost identical under these reaction conditions. Absolute configuration of the product was then determined with single crystal X-ray analysis of ***ent*-2a** (Fig. 2). Thus, the optimal conditions were identified to be a two-step procedure with **L7** as the ligand.

We found a broad substrate scope with respect to R^1^ and R^2^ of cyclobutanol (Table 2). When R^2^ was a phenyl group, various cyclobutanols bearing *para*- and *meta*-substituted phenyl groups (R^1^) reacted well to give the desired products in moderate to satisfactory yields and high enantioselectivities (ee = 94–98%); the substitutions could be alkyl, methoxy, or fluoro groups (Table 2, entries 1–5). The 1-alkylated 2-alkylenecyclobutanols are suitable substrates as well, and the desired bicyclic products were obtained in moderate yields with excellent ee values (Table 2, entries 6, 7). The variation of substitutions on the alkylene was then briefly investigated, and to our delight arenes bearing electron-donating methyl and methoxy at either the *para* or *ortho* positions with various combinations of benzene substitutions (R^1^) were compatible with the reaction conditions (Table 2, entries 8–16). In addition, a chloro group on the *para* position of R^2^ was well tolerated (Table 2, entry 17). The reaction also proceeded well when a furyl group was employed (Table 2, entry 18).

**Table 2:** Scope studies: enantioselective cycloadditions^*a*^ ^,^^*b*^

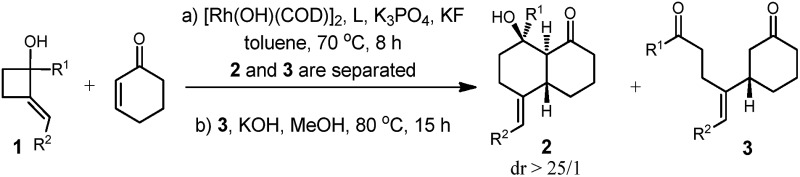						
Entry^*a*^	Product	R^1^	R^2^	**2**/**3**	Yield (**2**)^*b*^ [%]	ee^*c*^ [%]
1	**2b**	*p*-MeC_6_H_4_	Ph	1.1/1.0	55	98
2	**2c**	*p*-MeOC_6_H_4_	Ph	1.3/1.0	58	97
3	**2d**	*p*-FC_6_H_4_	Ph	1.9/1.0	62	97
4	**2e**	*m*-^i^PrC_6_H_4_	Ph	2.1/1.0	57	96
5	**2f**	*p*-^*n*^BuC_6_H_4_	Ph	1.7/1.0	56	94
6	**2g**	Me	Ph	2.0/1.0	58	97
7	**2h**	Et	Ph	2.7/1.0	58	97
8	**2i**	Ph	*p*-MeC_6_H_4_	2.8/1.0	57	95
9	**2j**	*p*-MeC_6_H_4_	*p*-MeC_6_H_4_	1.4/1.0	57	99
10	**2k**	*m*-ClC_6_H_4_	*p*-MeC_6_H_4_	2.6/1.0	66	96
11	**2l**	*p*-FC_6_H_4_	*p*-MeC_6_H_4_	3.8/1.0	77	92
12	**2m**	Ph	*o*-MeC_6_H_4_	2.1/1.0	57	98
13	**2n**	*p*-^*n*^BuC_6_H_4_	*o*-MeC_6_H_4_	4.4/1.0	56	99
14	**2o**	*m*-ClC_6_H_4_	*o*-MeC_6_H_4_	3.0/1.0	68	>99
15	**2p**	Ph	*p*-MeOC_6_H_4_	1.4/1.0	54	97
16	**2q**	*p*-MeC_6_H_4_	*p*-MeOC_6_H_4_	4.2/1.0	78	99
17	**2r**	*p*-MeC_6_H_4_	*p*-ClC_6_H_4_	3.6/1.0	78	99
18	**2r**	Ph	2-Fural	2.5/1.0	59	>99

^*a*^Unless otherwise noted, the two-step reactions were carried out under the optimized conditions (Table 1, entry 10).

^*b*^Combined yield of the two steps.

^*c*^The absolute configuration was assigned by analogy.

We then turned our attention to the variations of the α,β-unsaturated cyclic ketones. With **1a** as the substrate, a variety of cyclohex-2-enones was tested under optimal conditions. The reaction appeared to be highly sensitive to the electronic and steric properties of the substitutions. Substrates with a methyl substituent either on the double bond or at the β-position to the carbonyl group did not give the desired products. To our delight, 5,5-dimethylcyclohex-2-enone reacted under standard conditions to provide the desired product in moderate yield and with an excellent ee value as expected. We then investigated other cyclic enones with different ring sizes. For cyclopent-2-enone, the diketone could be obtained in good yield with an excellent ee value; however, no cyclized product was observed under a number of different conditions. The cyclohept-2-enone underwent annulation with **1a** under optimal conditions leading to the [4.5.0] bicyclic products in moderate yield with an excellent ee value. This represents another type of important molecular scaffold^11^ that is difficult to access using other methods (Table 3).

**Table 3:** Scope studies: enantioselective cycloaddition^*a*^

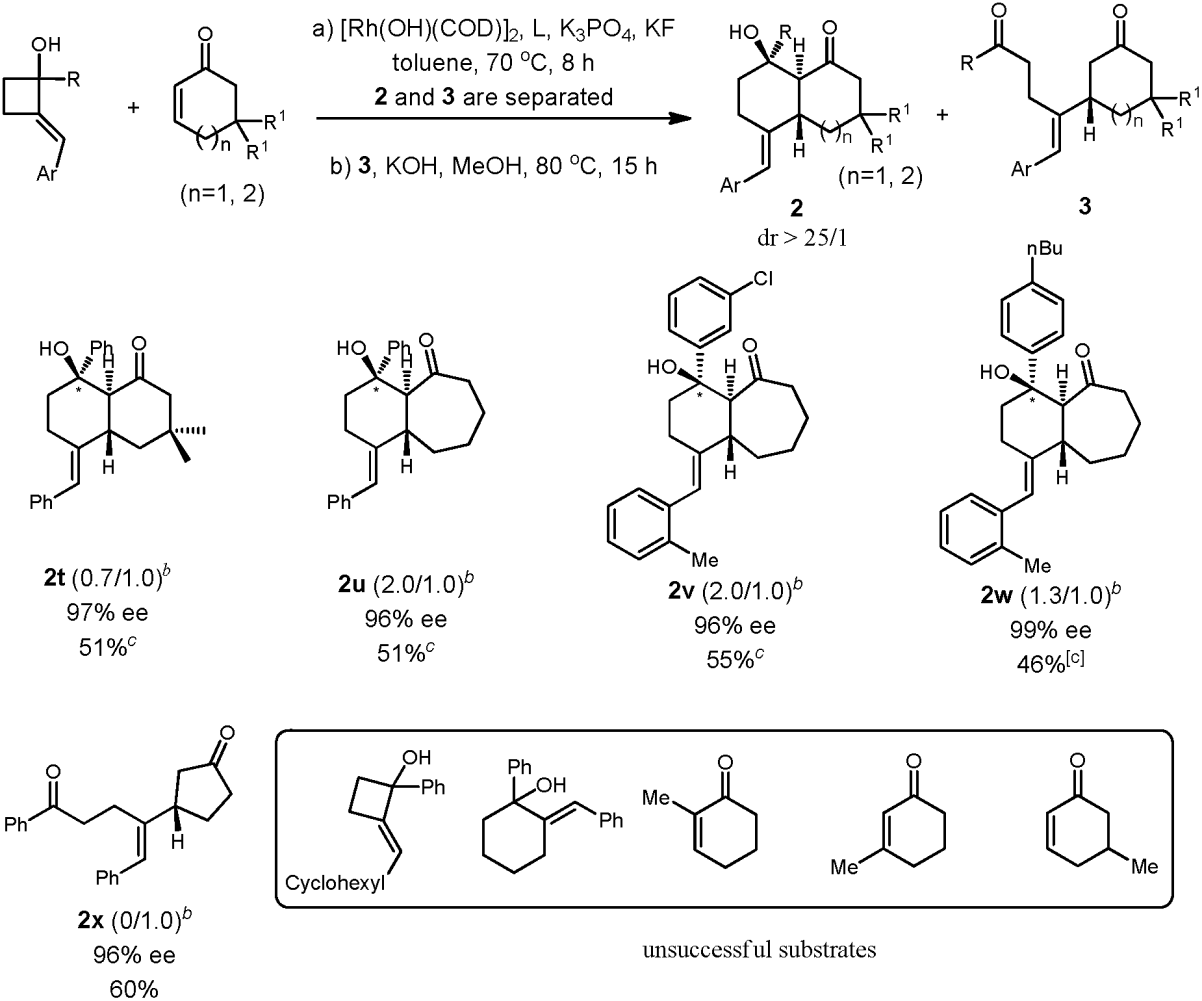

^*a*^Two-step reactions were carried out under the optimized conditions (Table 1, entry 10). The absolute configuration was assigned by analogy.

^*b*^Ratio of **2**/**3** in step a.

^*c*^Combined yield of **2** from the two steps.

**Scheme 1:**
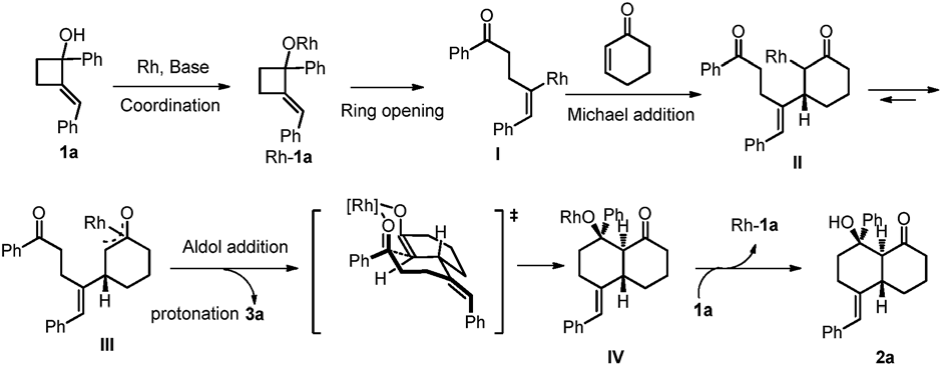
Proposed catalytic cycle.

According to previous studies and our observations,7f,j a stepwise reaction mechanism has been proposed (Scheme 1). At the start, a well-established rhodium(i) cyclobutanolate formation and β-carbon elimination occur to afford the vinylrhodium species **I**. A highly enantioselective Michael addition to the cyclohexanone occurs to form the intermediate **II** that undergoes isomerization to give the enolate **III**.^12^ The intramolecular aldol type cyclization proceeds in a highly stereoselective manner. Hydrolysis affords the final bicyclic product with regeneration of the catalyst. Concurrently, protonation of **III** is another pathway to yield the uncyclized product **3a**.

**Scheme 2:**
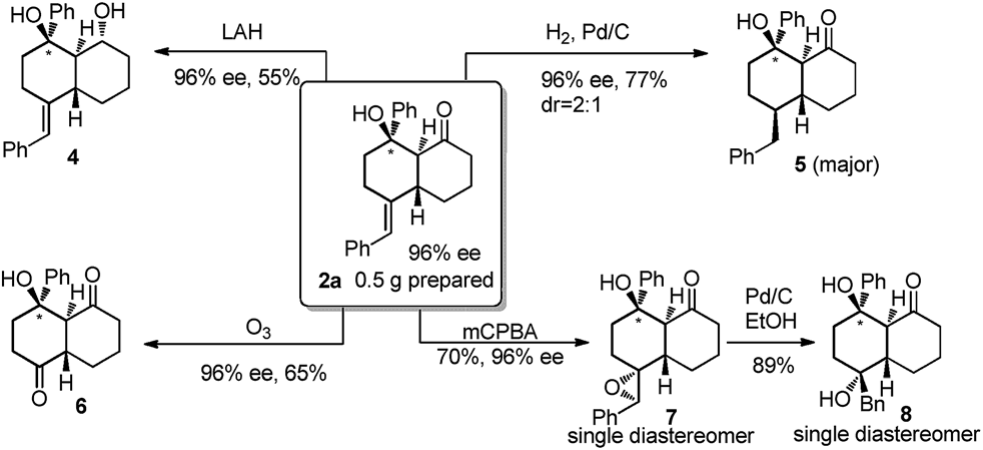
Synthetic utilities of *trans*-bicyclic products.

These series of bicyclic products are synthetically versatile building blocks due to the presence of several different functional groups for further elaborations (Scheme 2). For example, reduction of the ketone in **2a** using LiAlH_4_ produced the corresponding diol **4** in 55% yield as a single diastereomer. Reductive hydrogenation of the *exo* alkene gave rise to **5** as two inseparable diastereomers (2/1 ratio), both with four consecutive stereogenic centers. The double bond could be cleaved by ozonolysis leading to diketone **6** with a slightly decreased ee value. Epoxidation and the ring opening sequence proceeded effectively to afford both **7** and **8** as single diastereomers.

## Conclusions

In summary, we developed a rhodium(i)-catalyzed cycloaddition reaction of 2-alkylidene cyclobutanols with α,β-unsaturated cyclic ketones to form *trans*-bicyclic ketones containing three contiguous stereogenic centers in moderate yields with excellent enantioselectivities. Both [4.4.0] and [4.5.0] bicyclic systems are readily accessible in an optically pure form. The synthetic potential of the products was demonstrated *via* several easy derivatizations.

## Conflicts of interest

There are no conflicts to declare.

## Supplementary Material

Supplementary information

Crystal structure data
